# Posterior staphyloma is associated with the microvasculature and microstructure of myopic eyes

**DOI:** 10.1007/s00417-020-05057-0

**Published:** 2021-01-06

**Authors:** Fen Nie, Junyi Ouyang, Wenquan Tang, Lijia Luo, Mengdan Cao, Lurong Zhang, Dengming Zhou, Ke Liu, Daijin Ma, Xuanchu Duan

**Affiliations:** 1 Department of Ophthalmology, The Second Xiangya Hospital, Central South University, Changsha, Hunan China; 2 Aier School of Ophthalmology, Central South University, Changsha, Hunan China; 3 Aier Glaucoma Research Institute, Changsha Aier Eye Hospital, Changsha, Hunan China; 4 Department of Ophthalmology, Hunan Province Children’s Hospital, Changsha, Hunan China

**Keywords:** Posterior staphyloma, Myopia, Microvasculature, Microstructure

## Abstract

**Objective:**

To investigate the microvasculature and structural characteristics of the eyes of myopic patients and their association with posterior staphyloma (PS).

**Methods:**

This was a retrospective, case-control study comprising of 106 eyes from 72 individuals. Using 1:1 matching of axial length (AL) of their eyes, patients were allocated into a PS group or no posterior staphyloma (NPS) group. All patients were examined using ultra-widefield fundus imaging, optical coherence tomography angiography, and ocular biometry to acquire microvasculature and microstructure parameters.

**Results:**

The anterior chamber depth (ACD) of the PS group was significantly different from that of the NPS group (3.56 mm vs 3.76 mm, *P* < 0.001), as was 1ens thickness (3.72 mm vs 3.57 mm, *P* = 0.005) and spherical equivalent (SE)(-10.11D vs -8.80D, *P* = 0.014). The PS group had reduced choriocapillaris flow, subfoveal choroidal thickness (SFCT), and a thinner retinal layer compared with the NPS group. No difference in retinal blood flow between the two groups was observed. The PS group exhibited a smaller disc area (15082.89 vs 17,043.32, *P* = 0.003) and angle α between temporal retinal arterial vascular arcades (113.29°vs 128.39°, *P* = 0.003), a larger disc tilt ratio (1.41 vs 1.24, *P* < 0.001) and parapapillary atrophy (PPA) area (13840.98 vs 8753.86, *P* = 0.020), compared with the NPS group. Multivariate regression analysis indicated that disc tilt ratio (*P* = 0.031) and SFCT (*P* = 0.015) were significant predictors of PS. In addition, PS (*P* = 0.049), AL (*P* = 0.003), corneal refractive power (*P* < 0.001), ACD (*P* = 0.022), relative lens position (*P* = 0.045), and disc area (*P* = 0.011) were significant predictors of SE.

**Conclusions:**

PS was found to be closely linked to a reduction in choriocapillaris perfusion and anatomical abnormalities including posterior and anterior segments. Furthermore, PS exacerbated the progression of myopia.

**Supplementary Information:**

The online version contains supplementary material available at 10.1007/s00417-020-05057-0.

## Introduction

Posterior staphyloma (PS) is a hallmark abnormality of the ocular globe combined with pathological myopia (PM) [1–3]. It has been defined as the ectasia of a limited portion of the scleral wall with a radius shorter than the radius of the curvature of the surrounding area [3]. However, PS has also been reported in non-highly myopic eyes [4–6]. Due to its close correlation with the causal factors of blindness (i.e., macular hole, myopic retinoschisis, choroidal neovascularization, or retinal detachment), PS has recently been the subject of considerable attention.

No effective treatment is as yet available for PS, and its pathogenesis is highly controversial. Traditionally, the sclera has long been regarded as the tissue principally involved in the formation of PS. However, Ohno-Matsui et al. [6, 7] have proposed that the choroid or other tissues are potentially primarily involved in PS formation, based on previous studies. A number of investigations have shown significant differences in morphological features and vision between eyes with and without PS [8–10]. However, where axial length (AL) differs between two eyes, it is difficult to determine whether the anatomical abnormalities are due to AL itself or PS. In addition, whether PS is associated with decreased perfusion remains an open question. Fortunately, optical coherence tomography (OCT) angiography has developed as a practical non-invasive imaging technology allowing retinal and choriocapillaris microcirculation to be evaluated and quantified in vivo. Therefore, a 1:1 case-matched case-control study design was used here. The control group was matched for AL, which is a distinction of the present study compared with previous investigations. The purpose of the study was to investigate microvasculature- associated structural characteristics in patients with PS and to establish a comparison with AL-matched patients without PS. Furthermore, establishing a relationship between PS and increased myopia was an additional aim.

## Patients and methods

The present study adhered to the principles of the Declaration of Helsinki and was approved by the Ethics Committee of Changsha Aier Eye Hospital, Central South University, Changsha, China. The medical records of 445 consecutive patients with myopia presenting at the Refractive Department of Changsha Aier Eye Hospital from February 2019 to August 2019 were reviewed retrospectively. A comprehensive combination of examinations, including slit-lamp biomicroscopy, mydriatic indirect ophthalmoscopy, intraocular pressure (IOP), ultra-widefield fundus imaging (Optos), B-ultrasound, OCT angiography (RTVue-XR Avanti; Optovue, Fremont, CA, USA), best-corrected visual acuity, and refractive error, were conducted for all participants. Corneal refractive power, anterior chamber depth (ACD), lens thickness (LT), and AL were measured using a device based on unique swept-source biometry (IOL Master 700; Carl Zeiss Meditec, Germany). In addition, anterior segment length (ASL), 1ens position (LP), and relative lens position (RLP) were recorded for each patient (ASL = ACD + LT, LP = ACD +1/2LT, and RLP = LP/AL) [11].

**Fig. 1:**
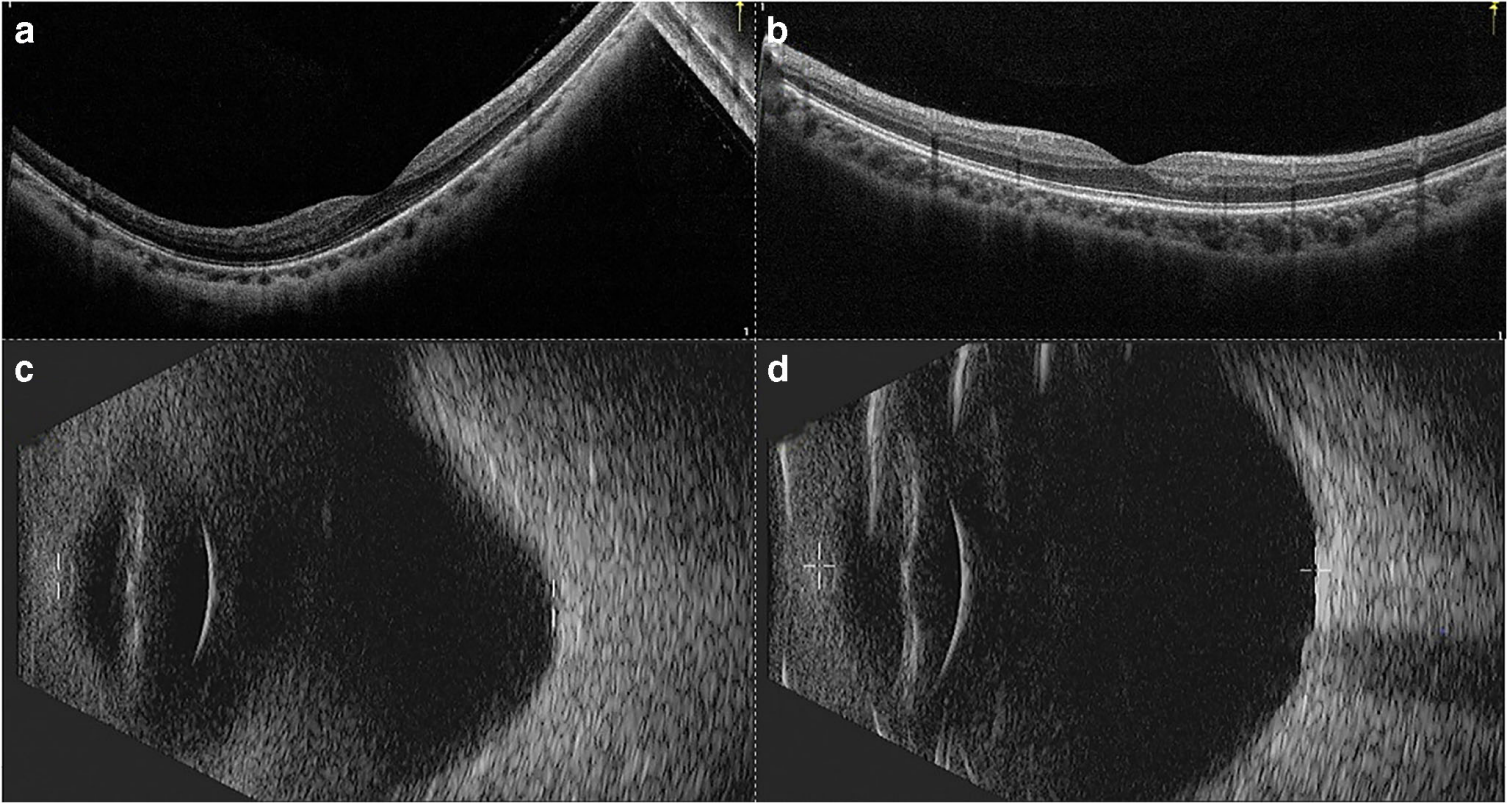
OCT and ultrasonographic images of PS and NPS. **a,c** An eye with an axial length of 26.76 mm that has PS. **b,d** An eye with an axial length of 26.76 mm without PS

Because of the challenge in identifying PS using three-dimensional magnetic resonance imaging (3D-MRI) or ultra-widefield OCT, and taking account of the inevitable subjectivity of examiners, patients were examined for PS by two experienced retinal ophthalmologists using OCT and ultrasonographic imaging. Depending on the presence or absence of PS and using 1:1 matching methodology, the patients were allocated into either the PS group or no posterior staphyloma (NPS) group. Eyes in the NPS group were matched for AL (± 0.2 mm). A matching example is presented in Fig. 1.

All myopic patients entered into the study satisfied the following inclusion criteria: aged between 18 and 50 years; AL > 24.0 mm; and IOP < 21 mm Hg. Patients older than 50 years were excluded since lenticular changes might have an impacted myopic refractive error. Exclusion criteria for the study were patients with a history of systemic or ocular disease, including hypertension, diabetes, hematologic disorders, previous ocular surgery, or other evidence of retinal pathology. In general, except for the optic disc, peripapillary, or retinal changes associated with myopia, no patient had other ocular abnormalities. Both eyes of each patient were assessed independently for recruitment into the study against the inclusion criteria.

### OCT angiography image acquisition and analysis

**Fig. 2:**
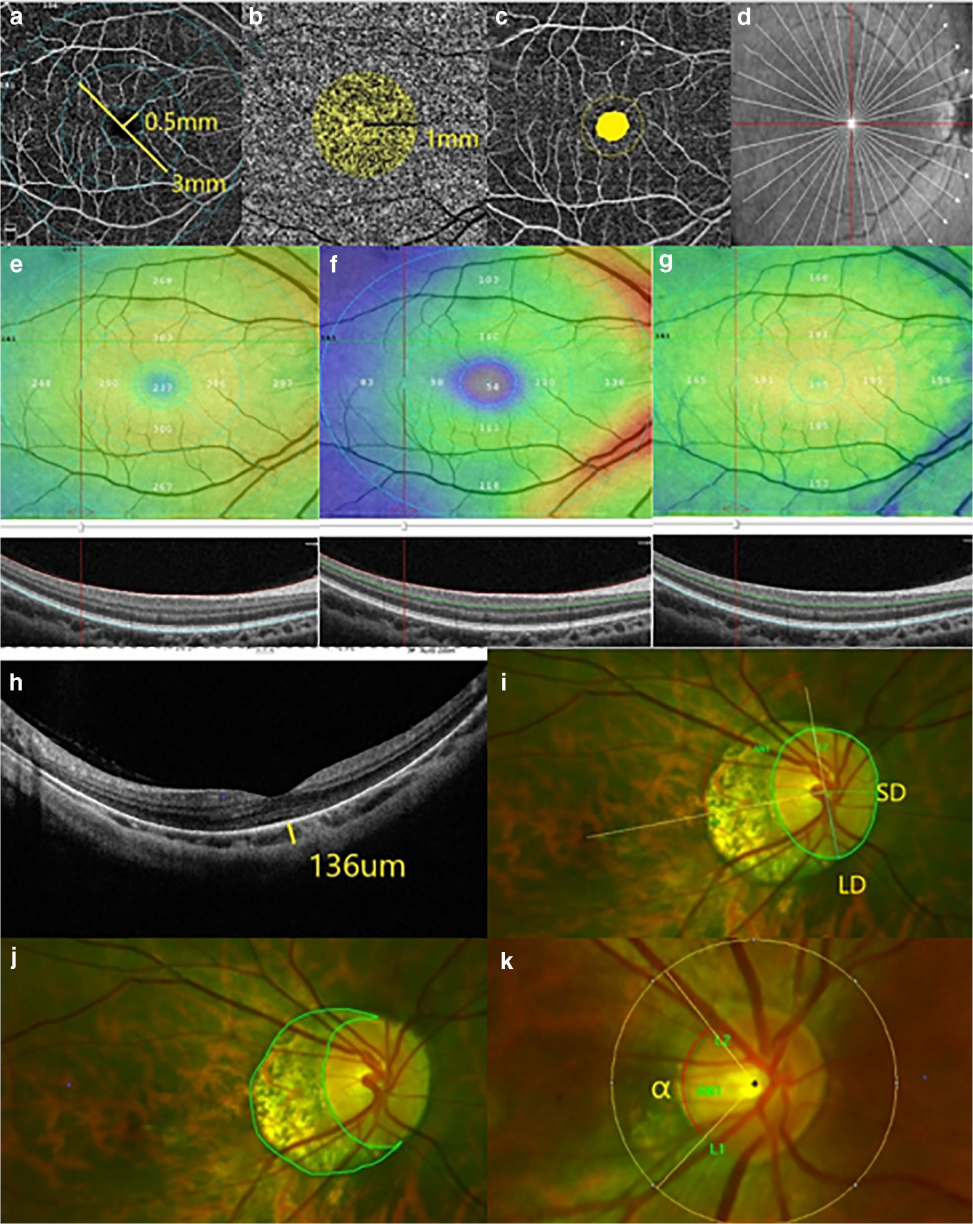
Measurement of vascular and structural parameters. **a** Fovea and parafovea vascular density (central circle of 0.5 mm radius, around the fovea with inner and outer diameters of 1 mm and 3 mm, respectively). **b** Choriocapillaris flow area (central circle of 1 mm radius). **c** FAZ area. **d** Eighteen 9-mm radial OCT scans. **e** Subfoveal retinal thickness was segmented from the internal limiting membrane to the retinal pigment epithelium. **f** The inner retina was segmented from the internal limiting membrane to the outer portion of the inner plexiform layer. **g** The outer retina was segmented from the outer portion of the inner plexiform layer to the outer portion of the hyperreflective line corresponding to the retinal pigment epithelium. **h** SFCT was measured manually. **i** The tilt ratio was defined as the ratio of the longest diameter (LD) to the shortest diameter (SD) of the optic disc. The degree of rotation was measured between the LD and the vertical meridian identified as a vertical line 90°from the horizontal line connecting the fovea and the center of the optic disc. **j** The area of peripapillary atrophy was outlined manually. **k** The angle α between temporal retinal arterial vascular arcades was measured from the center of the optic disc with a 250-pixel radius

The device used an 840-nm (bandwidth of 45 nm) light source with an A-scan rate of 70,000 scans per second. Cube scans of 6 × 6 mm and 4.5 × 4.5 mm centered on the fovea and optic disc, respectively, were acquired containing 400 × 400 A-scans. The mean retinal nerve fiber layer (RNFL) thickness was measured within a 3.45-mm-diameter circle, the center of which was positioned at the optic disc center. The ganglion cell complex (GCC) thickness was measured from the scan pattern, which covered a 7 × 7 mm scan area centered at the fovea. Eighteen 9-mm radial OCT scans were performed to evaluate the shape of the fundus. Major automated outcome measures recorded included vascular density (superficial retinal capillary, deep retinal capillary, and radial peripapillary capillary RPC) and retinal thickness (subfoveal retinal thickness, inner and outer retinal thickness in the parafovea): fovea (central circle with a 0.5-mm radius) and parafovea (the ring around the fovea with inner and outer diameters of 1 mm and 3 mm, respectively). The sizes of the foveal avascular zone (FAZ), acircularity index (AI), outer retinal flow area (central circle with a 1-mm radius), and choriocapillaris flow area (central circle with a 1-mm radius) were also obtained. In addition, the subfoveal choroidal thickness (SFCT) was measured manually. Measurements of the various parameters are illustrated in Fig. 2a–h in detail.

### Measurements of optic disc area, tilt, rotation, parapapillary atrophy (PPA) area, and angle α between retinal arterial vascular arcades

Images of the retina were captured using an ultra-widefield fundus (Optos) and then imported into Image-Pro Plus version 6.0 image processing software. Optic disc area, tilt, rotation, PPA area, and angle α were measured from the images by experienced retinal ophthalmologists (Fig. 2i–k). Mean values were used in the final analysis. The definitions of optic disc tilt and rotation and their measurements defined previously [12, 13] were adopted. Briefly, optic disc tilt was confirmed by tilt ratio, defined as the ratio between the longest and shortest diameters of the optic disc. The optic disc was considered a tilted disc where the tilt ratio exceeded 1.30. Optic disc rotation was defined as a deviation of the long axis of the optic disc from the vertical meridian, defined as a vertical line at 90°to a horizontal line connecting the fovea and center of the optic disc. The optic disc was considered rotated when the degree of rotation was more than 15°. A positive value represented an inferotemporal rotation, while, conversely, a negative value indicated a supranasal rotation. The value of PPA area (an inner crescent of chorioretinal atrophy with visible sclera and choroidal vessels) was expressed as the total number of pixels, measured using Image-Pro Plus software. The angle α between the temporal retinal arterial vascular arcades was measured from the center of the optic disc using a radius of 250 pixels.

### Statistical analysis

SPSS version 25.0 software (SPSS Inc., Chicago, IL) was used for all statistical analyses. The normality of distribution was assessed using a Kolmogorov-Smirnov normality test. An independent t-test, Mann-Whitney *U* test, and Chi-square test were used to assess differences between groups as appropriate and applicable. Spearman’s correlation coefficients were used to investigate the association between the ocular variables with SFCT and spherical equivalent (SE). Multivariate logistic regression analyses and multivariate linear regression analyses were used to identify independent variables associated with PS and SE, respectively. All variables with a *P* value of < 0.10 in a univariate analysis were included in the multivariate model. Both the unstandardized coefficient (*B*) and the standardized regression coefficient (*β*) were calculated using multivariable linear regression models. *P* values of < 0.05 were considered statistically significant.

## Results

A total of 106 eyes (72 patients) that satisfied the inclusion criteria were analyzed. Of these, 53 eyes were myopes with PS, and 53 were myopes without PS. As shown in Table 1, no significant differences in AL or corneal refractive power were identified between the two groups. Compared with the mean age in the PS group (27.38 ± 8.11 years), patients in the NPS group were significantly younger (23.51 ± 4.80 years) (*P* = 0.028). There was a significant difference in the ratio of genders in the PS group (6:27) compared with the NPS group (19:20) (*P* = 0.007). Unexpectedly, even though AL was matched, the SE of eyes with PS (-10.11 ± 2.66D) was higher than those without PS (-8.80 ± 2.04D) (*P* = 0.014).In addition, the PS group displayed a shallower ACD and thicker lens than the NPS group (*P* < 0.001 and *P* = 0.005, respectively). The mean ± standard deviation values of ACD in the PS and NPS groups were 3.56 ± 0.28 mm and 3.76 ± 0.28 mm, respectively, and 3.72 ± 0.27 mm and 3.57 ± 0.24 mm for LT, respectively. Furthermore, ASL (7.28 ± 0.28 mm vs 7.34 ± 0.22 mm), LP (5.42 ± 0.25 mm vs 5.55 ± 0.22 mm), and RLP (0.19 ± 0.01 vs 0.20 ± 0.01) exhibited statistical differences between the two groups (*P* = 0.022, *P* = 0.001, and *P* = 0.002).

**Table 1:** Demographic data, axial length, refractive error, and anterior segment parameters of PS group and NPS group

Variables	PS group (*n* = 53)	NPS group (*n* = 53)	*P* value
Age (years)	27.38 ± 8.11	23.51 ± 4.80	**0.028**^**‡**^
Gender (male:female)	6:27	19:20	**0.007**^**†**^
SE (D)	-10.11 ± 2.66	-8.80 ± 2.04	**0.014**^**‡**^
AL (mm)	27.29 ± 1.13	27.30 ± 1.14	0.950^*^
Corneal refractive power (D)	43.48 ± 1.75	43.19 ± 1.23	0.333^*^
ACD (mm)	3.56 ± 0.28	3.76 ± 0.28	< 0.001^*^
LT (mm)	3.72 ± 0.27	3.57 ± 0.24	0.005^*^
ASL (mm)	7.28 ± 0.28	7.34 ± 0.22	**0.022**^**‡**^
LP (mm)	5.42 ± 0.25	5.55 ± 0.22	**0.001**^**‡**^
RLP	0.19 ± 0.01	0.20 ± 0.01	**0.002**^**‡**^

*SE* spherical equivalent, *AL* axial length, *ACD* anterior chamber depth, *LT* lens thickness, *ASL* anterior segment length, *LP* 1ens position, *RLP* relative lens position. Factors with statistical significance are shown in boldface.^*^Independent *t*-test. ^†^Chi-square test. ^‡^Mann-Whitney *U* test

The vascular parameters measured by OCT angiography were compared between the groups (Table 2). No significant differences in foveal vascular density (superficial), parafoveal vascular density (superficial and deep), FAZ, AI, outer retina flow area, or RPC were identified between the two groups. Vascular density in the deep fovea (0.39% ± 0.06% vs 0.36% ± 0.07%) (*P* = 0.017) and choriocapillaris flow area (2.13 ± 0.11mm^2^ vs 2.05 ± 0.13mm^2^) (*P* < 0.001) were significantly higher in the NPS group than in the PS group.

**Table 2:** Retina, choroid, and optic disc vascular parameters of PS group and NPS group

Variables	PS group (*n* = 53)	NPS group (*n* = 53)	*P* value
Superficial vascular density (%)			
Fovea	0.20 ± 0.07	0.22 ± 0.05	0.103^*^
Parafovea	0.51 ± 0.04	0.51 ± 0.04	0.430^*^
Deep vascular density (%)			
Fovea	0.36 ± 0.07	0.39 ± 0.06	0.017^*^
Parafovea	0.53 ± 0.04	0.54 ± 0.05	0.578^*^
FAZ (mm^2^)	0.29 ± 0.10	0.25 ± 0.07	0.072^*^
AI	1.09 ± 0.03	1.08 ± 0.03	0.623^†^
Outer retina flow area (mm^2^)	1.06 ± 0.56	1.08 ± 0.44	0.600^†^
Choriocapillaris flow area (mm^2^)	2.05 ± 0.13	2.13 ± 0.11	< 0.001^*^
RPC (%)	0.49 ± 0.04	0.50 ± 0.03	0.139^*^

*FAZ* foveal avascular zone, *AI* acircularity index, *RPC* radial peripapillary capillary. Factors with statistical significance are shown in boldface. ^*^Independent *t*-test. ^†^Mann-Whitney *U* test

Structural parameters were compared between the groups (Table 3). Compared with myopic eyes without PS, the mean subfoveal retinal thickness (239.79 ± 18.45 μm vs 249.11 ± 17.54 μm) (*P* = 0.009), parafoveal inner retinal thickness (107.13 ± 6.32 μm vs 109.58 ± 5.35 μm) (*P* = 0.034), parafoveal outer retinal thickness (199.74 ± 7.61 μm vs 205.86 ± 9.07 μm) (*P* < 0.001), and SFCT (125.37 ± 70.44 μm vs 191.44 ± 59.15 μm) (*P* < 0.001) were significantly thinner in eyes in the PS group. Moreover, disc area (15,082.89 ± 5574.67 vs 17,043.32 ± 3768.24) (*P* = 0.003) and angle α (113.29 ± 18.35°vs 128.39 ± 27.10°) (*P* = 0.003) were smaller and the tilt ratio (1.41 ± 0.19 vs 1.24 ± 0.10) (*P* < 0.001) and PPA area (13,840.98 ± 10,769.85 vs 8753.86 ± 5491.09) (*P* = 0.020) larger in the PS group. Nevertheless, no significant differences in the degree of disc rotation, mean RNFL thickness, or GCC thickness were identified.

**Table 3:** Retina, choroid, and optic disc structure parameters of PS group and NPS group

Variables	PS group (*n* = 53)	NPS group (*n* = 53)	*P* value
Subfoveal retinal thickness (μm)	239.79 ± 18.45	249.11 ± 17.54	0.009^*^
Parafoveal inner retina thickness (μm)	107.13 ± 6.32	109.58 ± 5.35	0.034^*^
Parafoveal outer retina thickness (μm)	199.74 ± 7.61	205.86 ± 9.07	< 0.001^*^
SFCT (μm)	125.37 ± 70.44	191.44 ± 59.15	**< 0.001**^**‡**^
Optic disc area	15,082.89 ± 5574.67 17,043.32 ± 3768.24	**0.003**^**‡**^	
Optic disc tilt			
Tilted disc, *n* (%)	34(64.2)	12(22.6)	**< 0.001**^**†**^
Tilt ratio	1.41 ± 0.19	1.24 ± 0.10	< 0.001^*^
Optic disc rotation			
Rotation disc, *n* (%)	36(67.9)	38(71.7)	0.672^†^
Rotation degree (°)	9.93 ± 27.88	-1.47 ± 15.16	0.380^‡^
PPA	13840.98 ± 10,769.85	8753.86 ± 5491.09	**0.020**^**‡**^
Angle α(°)	113.29 ± 18.35	128.39 ± 27.10	**0.003**^**‡**^
Average RNFL thickness (μm)	101.23 ± 8.98	98.59 ± 7.14	0.163^‡^
GCC thickness (μm)	92.35 ± 19.43	94.85 ± 4.56	0.580^‡^

*SFCT* subfoveal choroidal thickness, *PPA* parapapillary atrophy, *RNFL* retinal nerve fiber layer, *GCC* ganglion cell complex. Factors with statistical significance are shown in boldface. ^*^Independent *t*-test. ^†^Chi-square test. ^‡^Mann-Whitney *U* test

**Fig. 3:**
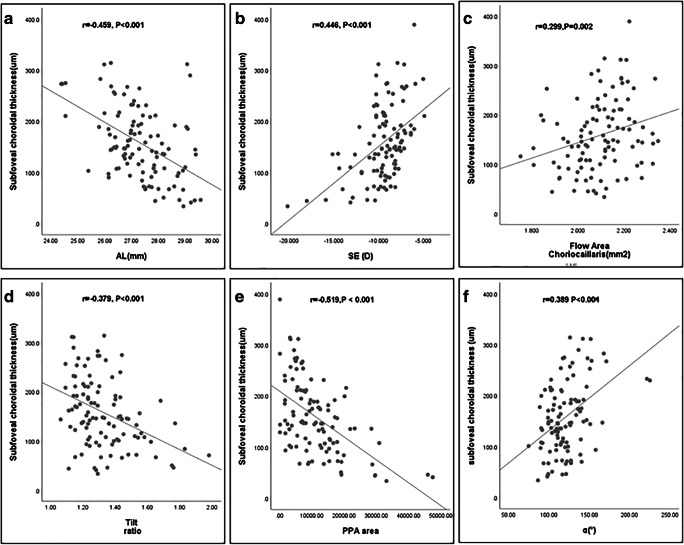
Scatterplots of the relationships between SFCT and **a** AL, **b** SE, **c** choriocapillaris flow area, **d** tilt ratio, **e** PPA area, and **f** angle α

Linear correlation analysis between SFCT and ocular parameters is summarized in Fig. 3. While SFCT was negatively associated with PPA area (*r* = -0.519,*P* < 0.001), and to a lesser degree with AL (*r* = -0.459, *P* < 0.001) and tilt ratio (*r* = -0.379, *P* < 0.001), it was positively associated with SE (*r* = 0.446, *P* < 0.001), choriocapillaris flow area (*r* = 0.299,*P* = 0.002), and angle α (*r* = 0.389,*P* < 0.001).

Table 4 displays the relationships between the PS and various ocular parameters. Univariate regression analysis demonstrated a significant correlation of PS with age (*P* = 0.031), gender (*P* < 0.001), SE (*P* = 0.031), choriocapillaris flow area (*P* < 0.001), ACD (*P* = 0.002), LT (*P* = 0.037), optic disc area (*P* = 0.055), tilt ratio (*P* < 0.001), PPA area (*P* = 0.032), and angle α(*P* = 0.007). Multivariate logistic regression modeling indicated that tilt ratio (*P* = 0.031) and SFCT (*P* = 0.015) were significant predictors of PS.

**Table 4:** Logistic regression analysis with the dependent variable being PS

Variables	*B*	Univariate Standard error	*P* value	*B*	Multivariate Standard error	*P* value
Age(years)	0.082	0.038	0.031	0.095	0.079	0.225
Gender (male:female)	1.738	0.478	< 0.001	0.076	0.815	0.926
SE(D)	-0.212	0.098	0.031	0.094	0.180	0.600
Choriocapillaris flow area (um^2^) 0.001	-6.573	1.932	-2.653	2.801	0.344	
ACD (mm)	-2.396	0.767	0.091	-2.801	1.445	0.052
LT (mm)	1.903	0.911	0.037	-0.495	2.039	0.808
Optic disc area	< 0.001	< 0.001	0.055	< 0.001	<0.001	0.085
Tilt ratio	8.184	1.941	< 0.001	**5.718**	**2.649**	**0.031**
PPA area	< 0.001	< 0.001	0.032	< 0.001	< 0.001	0.902
Angle α (°)	-0.029	0.011	0.007	-0.023	0.020	0.241
subfoveal retinal thickness (um)	-0.030	0.012	0.012	-0.012	2.801	0.344
SFCT (um)	-0.015	0.004	< 0.001	**-0.016**	**0.006**	**0.015**

*B* unstandardized coefficient, *SE* spherical equivalent, *PPA* parapapillary atrophy. Factors with statistical significance are shown in boldface. Variables with a *P* value of < 0.10 in the univariate analysis were included in the multivariate model

**Fig. 4:**
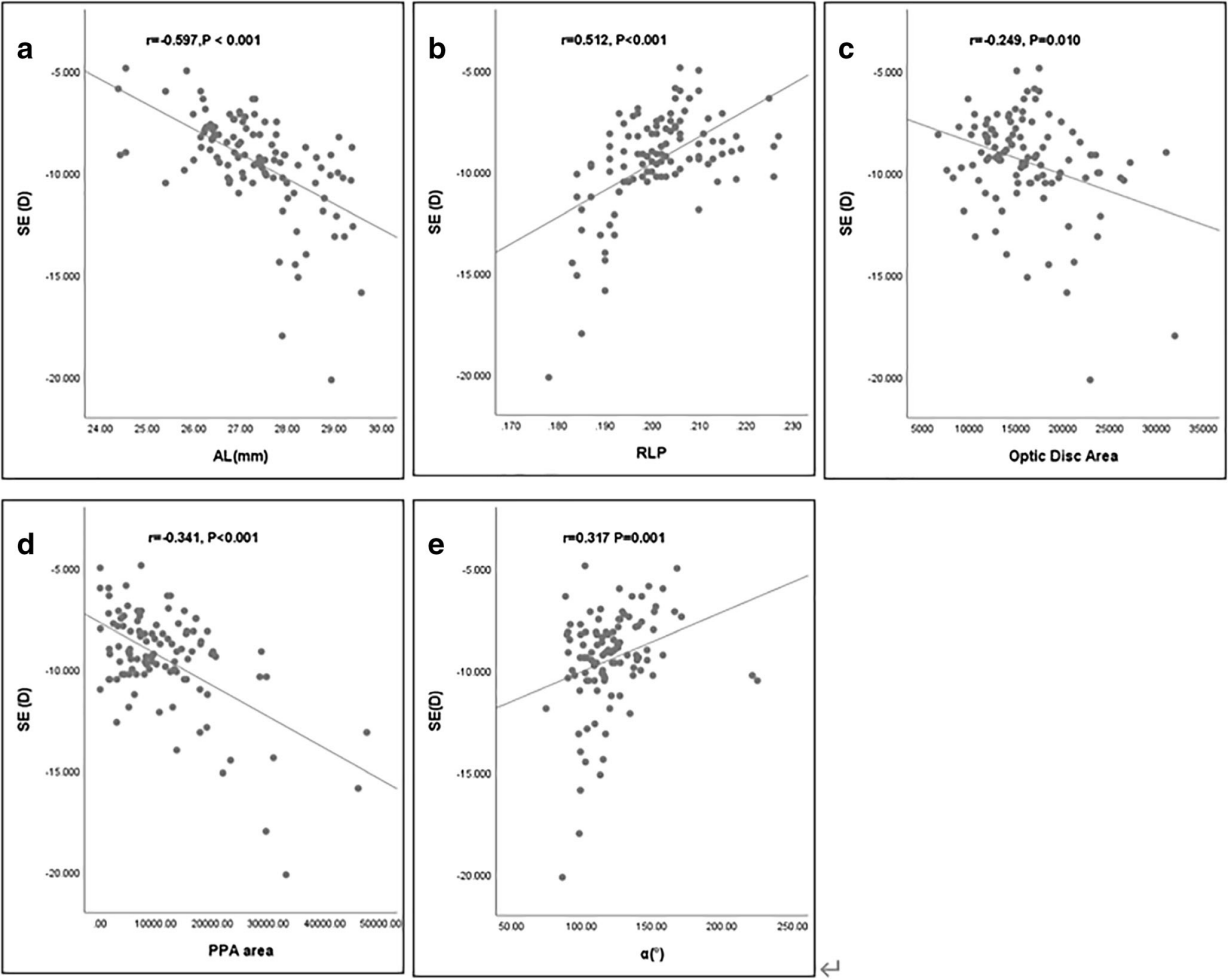
Scatter plots demonstrating the relationships between SE and **a** AL, **b** RNP, **c** optic disc area, **d** PPA area, and **e** angle α

Linear correlation analysis between SE and ocular parameters are shown in Fig. 4. SE was negatively associated with AL (*r* = -0.597, *P* < 0.001), optic disc area (*r* = -0.249, *P* = 0.010), and PPA area (*r* = -0.341, *P* < 0.001) and positively associated with RLP (*r* = 0.512, *P* < 0.001) and angle α (*r* = 0.317, *P* = 0.001).

Table 5 displays the relationships between the SE and a number of ocular variables. Univariate regression analysis indicated a significant correlation of SE with PS (*P* = 0.005), age (*P* = 0.005), AL (*P* < 0.001), corneal refractive power (*P* < 0.002), ACD (*P* = 0.054), LT (*P* = 0.032), RLP (*P* < 0.001), optic disc area (*P* < 0.001), PPA area (*P* < 0.001), angle α (*P* = 0.003), and SFCT (*P* < 0.001). Multivariate linear regression modeling suggested that PS (*P* = 0.049), AL (*P* = 0.003), corneal refractive power (*P* < 0.001), ACD (*P* = 0.022), RLP(*P* = 0.045), and optic disc area (*P* = 0.011) are significant predictors of SE.

**Table 5:** Linear regression analysis to determine the correlation between variables and the SE

Variables	Univariate			Multivariate		
	*B*	*β*	*P* value	*B*	*B*	*P* value
PS (vs without PS)	-1.308	-0.269	0.005	**-0.625**	**-0.128**	**0.049**
Age (years)	-0.099	-0.271	0.005	0.028	0.076	0.377
AL (mm)	-1.224	-0.566	< 0.001	**-5.302**	**-2.450**	**0.003**
Corneal refractive power (D)	-0.490	-0.303	0.002	**-0.896**	**0.553**	**< 0.001**
ACD (mm)	1.553	0.188	0.054	**20.297**	**2.453**	**0.022**
LT (mm)	-1.924	-0.209	0.032	8.399	0.912	0.061
RLP	131.422	0.546	<0.001	**-484.241**	**-2.012**	**0.045**
Optic disc area	< 0.001	-0.322	< 0.001	**-7.511 × 10**^**-5**^	**-0.148**	**0.011**
PPA area	< 0.001	-0.564	< 0.001	1.835 × 10^-5^	0.067	0.384
Angle α	0.029	0.290	0.003	0.009	0.091	0.127
SFCT (um)	0.016	0.485	< 0.001	< 0.001	0.008	0.913

*B* unstandardized coefficient, *β* standardized coefficient, *SE* spherical equivalent, *AL* axial length, *ACD* anterior chamber depth, *LT* lens thickness, *ASL* anterior segment length, *LP* 1ens position, *RLP* relative lens position, *PPA* parapapillary atrophy, *SFCT* subfoveal choroidal thickness. Factors with statistical significance are shown in bold. Variables with a *P* value of < 0.10 in the univariate analysis were included in the multivariate model

## Discussion

The principal purpose of the present study was to investigate whether PS is associated with the microvascular and structural ocular integrity through comparisons of eyeballs with matching AL. Corroborating previous findings [9, 14], PS patients in the present study were significantly older at 27.38 ± 8.11 years than those in the NPS group (23.51 ± 4.80 years). The number of female patients was greater than those that were male in the PS group. This observation suggests that gender may be a risk factor for PS. It has already been confirmed that age and AL are such risk factors [7]. However, age and AL were excluded as predictors of PS in the logistic regression model as a result of matching AL and subtle difference in age between the two groups.

Surprisingly, the PS group exhibited greater myopic SE refractive error although the AL of the two groups was matched. Measurements of the anterior segments may help explain this phenomenon. From the results detailed in Table 1, we can speculate that PM with PS occurred with a shorter ASL, shallower ACD, and thicker LT compared with eyes in the NPS group. Moreover, the LP and RLP were anatomically further forward. The logical relationship can be explained as a relative forward movement of the lens position leading to a shorter ASL and thickening of the lens resulting in a relatively shallower ACD due to the presence of PS, which exacerbated myopia as a result of the positive association between SE and RLP (Fig. 4).In addition, multivariate linear regression analysis demonstrated that PS, AL, corneal refractive power, ACD, and RLP were significant predictors of SE. From the optical point of view, a 1-mm reduction in ACD (such as when the lens moves forward) would increase the refractive power of the eye by 1.4D, assuming that other ocular factors remain constant [15]. More importantly, the curvature of the lens is closely linked to the refractive power, which may also have changed.

As outlined in Table 2, such observations suggest that only deep foveal vascular density and choriocapillaris flow area were lower in the PS group. Given that FAZ and AI better represent blood flow within the foveal region, the principal structure with reduced vascular density in the macular area was the choroid, while no significant difference was found in the RPC which represents blood flow at the optic disc. The lower levels of significant retinal vascular density in younger individuals with PS is probably because it does not develop until later in life. When we closely tracked the parameters of the macular structure, we found that eyes with PS displayed a thinner retinal layer and SFCT. The mean SFCT measured in the present study was different from those reported previously. In a study reported by Zhou [9], SFCT in 48 eyes with PS with a mean age of 66.6 years was 54.9 μm, thinner than that observed here. However, the age difference between the studies may explain that inconsistency.

It is well established that retinal vascular density is negatively associated with AL [16–18]. In the present study, macular retinal thickness decreased, while retinal blood flow density in the PS group did not significantly change. This suggests that a compensatory increase in retinal blood flow in eyes with PS had occurred that preserved retinal function. A significant reduction in retinal vascular density in individuals with PS probably does not develop until blood flow decompensates. Therefore, the thickness/microvascular mismatch suggests that structural degeneration may occur faster than vascular damage in myopic eyes. In another study, Wu [19] recently demonstrated that hypoxia is a key modulator of scleral extracellular matrix remodeling as myopia progresses. Additionally, the antihypoxic drugs slowed down the progression of experimental myopia without affecting normal ocular growth in guinea pigs. Based on the aforementioned studies, the authors speculate that myopia-related visual signals lead to a decrease in choroidal perfusion, resulting in reduced levels of oxygen and a lack of nutrient supply to the neighboring avascular sclera. Scleral hypoxia thus triggers scleral myofibroblast transdifferentiation and altered scleral biomechanics. These changes contribute to scleral thinning and weakening, ultimately resulting in excessive axial elongation. Ohno-Matsui et al. [7] also proposed that the choroid is primarily involved in PS formation for similar reasons. In the current study, choroidal capillary perfusion in eyes with PS was significantly lower than in eyes without PS, with the possibility that deep choroidal blood flow was reduced. Moreover, logistic regression analysis suggested that SFCT and disc tilt ratio were factors related to the presence of PS, further supporting the hypothesis that the choroid is the primary tissue responsible for the formation of PS. However, this hypothesis does not explain why non-myopic eyes develop PS, such as in retinitis pigmentosa or toxoplasmosis with PS [4, 6]. The staphyloma edges in retinitis pigmentosa exhibit gradual choroidal thinning from the fundus periphery toward the edge of the staphyloma with a gradual re-thickening of the choroid towards the posterior pole, and a slight inward protrusion of the sclera at the border of the staphyloma. A similar characteristic of choroidal thickness has been observed previously in PM with PS [10, 20]. This observation implies that eyes with PS exhibit a unique attribute, independent of myopia.

In the present study, the association between PS and the characteristics of optic disc morphology has been outlined. Combined with the results detailed in Tables 3 and 4, a smaller optic disc and larger disc tilt ratio and PPA were found in the PS group, possibly representing predictors of asymmetric deformation of the eyeball at an early stage, especially the disc tilt ratio. The relationship between tilted disc and progression of glaucoma is poorly understood, especially regarding causal factors that impact it. However, it has been assumed that myopic eyes with a tilted disc reliably leads to stable progression of glaucoma compared with eyes that have a non-tilted disc after the elongation process is complete [21–23]. In other words, disease progression in myopic glaucoma patients with PS is more stable than those without PS. However, the area of the PPA β-zone is closely related to glaucoma progression [24, 25], Therefore, it is of vital importance to pay additional attention to the eyes of young myopic patients with PS at an early stage although we did not find significant differences in the degree of optic disc rotation and mean RNFL thickness in the present study. Jonas et al. [26] has already confirmed that axial elongation is correlated with a smaller angle of temporal arterial arcade, caused by an elongation of the distance between the disc and foveola. In the present research, the PS group displayed a smaller angle α between the temporal retinal arterial vascular arcades, suggesting that the position of the optic disc was dragged nasally due to the PS. Hence, in addition to elongation of the AL, PS also represents a risk factor for decreased angle of temporal arterial arcade. Radcliffe et al. [27] found that shifts in position of retinal blood vessels occurred in eyes with functionally progressive glaucoma. Therefore, the specific relationship between PS and progression of glaucoma deserves further study.

As the choroid is possibly the structure primarily involved in PS formation, it was important to conduct a linear correlation analysis between the SFCT and ocular parameters. Partially corroborating previous studies conducted by Chen [28], we found that thinning of the choroid may be closely correlated with elongation of its axis, a reduction in choroidal capillary perfusion, larger disc tilt ratio, enlargement of the PPA area, and reduction of temporal retinal arterial vascular arcades. Although the subjects in the present study did not have myopic maculopathy, it is extremely important to closely monitor them for the condition, especially those with large areas of PPA with implications of choroidal thinning and subsequent myopic maculopathy.

We acknowledge that there are a number of limitations associated with the present study. This was a retrospective study, and hence longitudinal data were not available. It is widely reported that age is a critical risk factor for PS and, thus, a longitudinal study to observe the development of PS is required. In the present study, the two groups were not matched well by age. Furthermore, the patient cohort comprised of individuals in hospital and not via population-based screening; thus, it may not be truly representative of the entire population. Lastly, there are limitations in diagnosing PS using B-ultrasound and OCT. Swept-source ultra-widefield OCT and 3D MRI may be better choices. As a result, we could not identify the type of PS associated with structural changes in the eyeball.

Consequently, PS was closely linked with reductions in choroidal perfusion and anatomical abnormalities, including posterior and anterior segments. It is not possible to regain normal vision once PS-related complications occur. Hence, there is an unmet need for preventive measures to avoid the development of myopia-associated complications in highly myopic eyes that require urgent care. Future work should focus on the etiology of PS.

## Supplementary Information

ESM 1 (XLSX 66 kb)

## Data Availability

The datasets used and analyzed for the present study are available from the corresponding author.
